# Comparison of a Subepithelial Connective Tissue Graft and a Xenogeneic Collagen Matrix in Combination with a Coronally Advanced Flap for Gingival Recession Coverage with 12-Month Follow-Up: A Systematic Review and Meta-Analysis

**DOI:** 10.3390/medicina61091596

**Published:** 2025-09-04

**Authors:** Alma Pranckevičienė, Ekaterina Chuiko, Inga Vaitkevičienė, Rugilė Anužytė, Vita Mačiulskienė-Visockienė

**Affiliations:** Departament of Dental and Oral Pathology, Faculty of Odontology, Lithuanian University of Health Sciences, Eiveniu 2, LT-50161 Kaunas, Lithuania; almapran1003@kmu.lt (A.P.); ekaterina.chuiko@stud.lsmu.lt (E.C.); inga.vaitkeviciene@lsmu.lt (I.V.); rugile.anuzyte@stud.lsmu.lt (R.A.)

**Keywords:** multiple recessions, coronally advanced flap, tunneling technique, connective tissue graft, xenogeneic matrix

## Abstract

*Background and Objectives*: This systematic review and meta-analysis aimed to evaluate the efficacy of xenogeneic collagen matrix (XCM) in combination with a coronally advanced flap (CAF) in the management of multiple gingival recessions and to compare its outcomes with those achieved using the conventional connective tissue graft (CTG) + CAF approach. *Materials and Methods*: After searching and reviewing the literature in the electronic databases PubMed/PMC, Google Scholar, ScienceDirect, Cochrane Library, and LILACS, 601 publications were found. The titles and abstracts of 543 publications were screened. After evaluating the full text of 290 publications, based on the inclusion criteria, four randomized controlled clinical trials were included in the systematic review and meta-analysis. In all the studies, the test group was treated with XCM + CAF, whereas in the control group, CTG + CAF was used. *Results*: Clinical attachment level (CAL), gingival recession depth (GR), keratinized tissue width (KTW), and complete root coverage (CRC) statistically significantly (*p* < 0.05) improved in both groups in all of the studies. Inter-group comparison showed better results in the control group in individual studies. All clinical trials reported a statistically significant (*p* < 0.05) decrease in surgery time and less postoperative pain in the test group. The meta-analysis of KTW (−0.438 [95% CI, −0.714 to −0.163], *p* < 0.002) and GR (0.35 [95% CI, 0.098 to 0.602], *p* < 0.001) showed a significant difference between the test and the control groups in all of the studies. CAL (0.78 [95% CI, −0.305 to 0.461], *p* > 0.05) showed no statistically significant difference between test and control groups. *Conclusions*: CTG + CAF remains the gold standard in root coverage procedures. However, XCM offers a less invasive alternative with improved patient comfort, less postoperative pain, shorter surgical time, and acceptable clinical outcomes.

## 1. Introduction

Gingival recession (GR) is defined as the apical displacement of the gingival margin relative to the cemento-enamel junction (CEJ), often leading to exposure of the root surface and associated clinical attachment loss [1]. Multiple etiological factors contribute to GR, including chronic trauma, periodontal disease, periodontal treatment, and occlusal trauma [2]. It is a highly prevalent condition: more than 50% of the population presents with one or more gingival recession sites of 1 mm or greater [3]. The prevalence and severity of GR increases with age. Notably, age is not directly associated with GR; it only increases the likelihood of the contributing factors acting on it [4,5]. Also, GR prevalence is different for males and females. The male population is more prone to GR than the female population [5].

Clinically, GR may lead to complications such as compromised esthetics, dentin hypersensitivity, root caries, and an increased risk of abrasion in the exposed root areas [6,7].

Due to its high prevalence and the increasing demand for esthetic and functional rehabilitation, several treatment options have been developed for GR management [8]. In cases of minimal, asymptomatic recession, non-surgical approaches may be applied. However, in more advanced cases, surgical interventions are required to restore the gingival anatomy in relation to the CEJ and to improve soft tissue thickness. Commonly used techniques include the coronally advanced flap (CAF) and the tunneling technique (TUN), often combined with a connective tissue graft [9,10]. The subepithelial connective tissue graft (CTG) in combination with CAF is considered the gold standard for achieving root surface coverage and enhancing the periodontal biotype. The CAF technique is described as coronal shift of the soft tissues on the exposed root surfaces achieved by vertical incisions. It provides a wide recipient bed and promotes high vascularization, thus making the healing process faster [11].

Despite its effectiveness, this technique is associated with limitations such as the need for a second surgical site, patient discomfort, intraoperative and postoperative complications, limited donor tissue availability, and prolonged surgical time [10,12].

To overcome these drawbacks and offer an alternative in cases where keratinized mucosa thickness is insufficient, several biomaterials have been introduced. The focus of our study was the use of xenogeneic collagen matrix (XCM), which has shown superior properties compared to other biomaterials [13,14]. XCM is a three-dimensional bilayer made of porcine collagen types I and III without cross-linking or chemical treatment and is clinically approved [12,15]. The bilayer consists of a thick, porous layer that stabilizes the blood clot and promotes tissue integration and angiogenesis, and a compact layer that accommodates sutures, enhances wound healing, and facilitates cell adhesion [14,16]. XCM has shown the potential to replace CTG, as it provides satisfactory esthetic outcomes, including color and texture scores comparable to those of CTG. Additionally, it eliminates patient morbidity and pain associated with CTG harvesting [17,18]. However, uncertainties remain regarding the clinical outcomes and the superiority of XCM over CTG.

This systematic review aimed to evaluate the efficacy of XCM in combination with CAF in the management of multiple gingival recessions and to compare its outcomes with those achieved using the conventional CTG + CAF approach.

## 2. Methods

### 2.1. Protocol and Registration

This systematic review was conducted following the Preferred Reporting Items for Systematic Reviews and Meta-Analyses (PRISMA) guidelines [19]. The study protocol was registered in the International Prospective Registry of Systematic Reviews (PROSPERO) under the registration number CRD420251006697 on 18 March 2025.

### 2.2. Focus Question

The focus question guiding this systematic review was: “Can XCM in combination with CAF replace CTG in the treatment of multiple gingival recessions?”. The PICOT (Population, Intervention, Comparison, Outcome, Time) framework was applied as outlined in Table 1 [20].

### 2.3. Outcome Variables

- Keratinized tissue width (KTW)
- Clinical attachment level (CAL)
- Gingival recession depth (GRD)
- Complete root coverage (CRC)
- Duration of surgery
- Patient-centered outcomes (esthetics, pain, and satisfaction)

### 2.4. Type of Publications and Studies

The review focused only on randomized controlled clinical trials (RCTs) that compared XCM + CAF with CTG + CAF, published in English between 2010 and 2025.

### 2.5. Information Source

The literature search was conducted using PubMed/PMC, Google Scholar, ScienceDirect, the Cochrane Library, and LILACS. All studies published up to 3 March 2025 were included. The electronic databases were analyzed by the investigators (A.P., E.C.) in accordance with the PRISMA guidelines [21].

### 2.6. Search Strategy

A detailed analysis of scientific electronic data was carried out by the independent reviewers (A.P., E.C.) using keywords and their combination, as shown in Table 2. The first (#1) search keywords were based on terms associated with xenogeneic collagen matrix biomaterial. The second (#2) search was related to connective tissue grafts. The third (#3) search focused on the surgical approach to the recession area. The fourth (#4) search was based on the type of periodontal problems to be treated, such as multiple recessions. The final search (#5) encompassed combinations of keywords in four earlier searches.

Various combinations of the Boolean operators “AND” and “OR” were used in these searches.

The search words were chosen, combining Medical Subject Headings (MeSH terms) as follows: ((((((“Periodontal Diseases”[Mesh]) AND (“Gingival Recession/surgery”[Mesh] OR “Gingival Recession/therapy”[Mesh])) AND “Autografts/transplantation”[Mesh])) AND “Heterografts/transplantation”[Mesh]) AND “Surgical Flaps”[Mesh]) AND “Humans”[Mesh].

### 2.7. Selection of Studies

Firstly, titles and abstracts were independently screened by the reviewers (A.P., E.C.) based on the predefined inclusion and exclusion criteria. Further on, full texts of potentially relevant studies were retrieved and assessed for eligibility. Any disagreements regarding the inclusion of the studies were resolved through discussion.

### 2.8. Inclusion Criteria

(a) Randomized controlled clinical trials on humans aged 18 and older.
(b) Use of XCM in the test group and CTG in the control group.
(c) Presentation of sufficient information (KTW, CAL, GR, CRC, surgery time, and patients’ outcomes) on the baseline and at the end of the study.
(d) Comparison of XCM + CAF (test group) vs. CTG + CAF (control group).
(e) Follow-up period of 12 months.
(f) Treatment performed on natural teeth with multiple recessions.
(g) Patients with periodontal and systemic health.
(h) At least 20 patients included in the study.

### 2.9. Exclusion Criteria

(a) In vitro studies, animal studies, reviews, systematic reviews, meta-analyses, PhD theses, and case reports.
(b) Articles not in English.
(c) Studies lacking comprehensive details.
(d) Follow-up period less than 12 months.
(e) Surgical technique other than CAF.
(f) Use of biomaterials other than XCM.
(g) Treatment performed on implants.
(h) Studies on pregnant or lactating women.
(i) Patients with prior recession treatment in the target area.
(j) Cervical restorations in the teeth under investigation (their presence could interfere with the predictability of the recession root coverage procedure).

**Figure 1:**
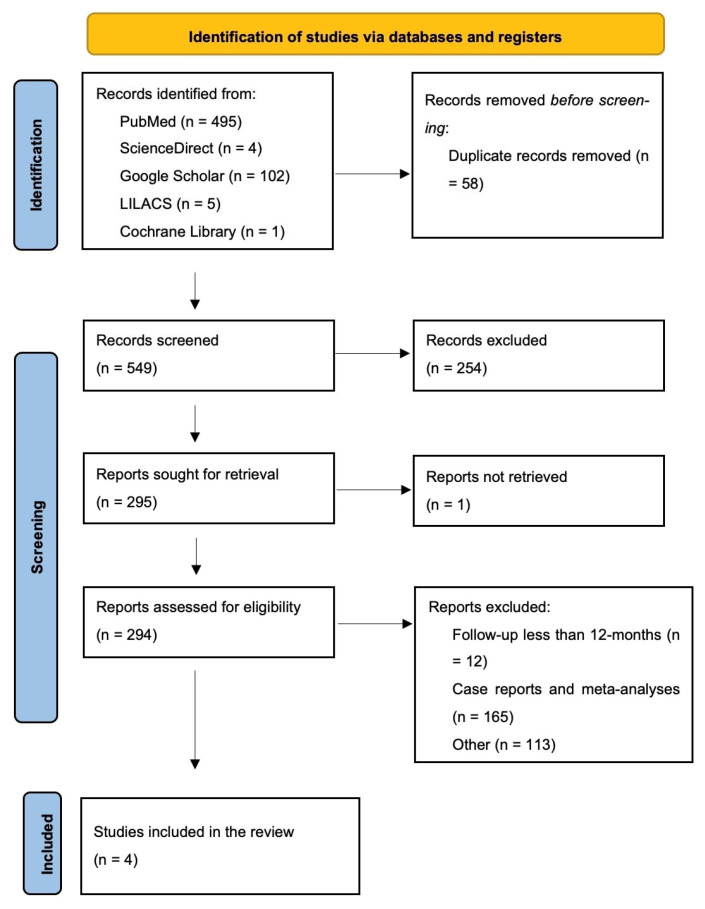
PRISMA flow diagram of study selection.

A total of four studies [15,22,23,24] met the inclusion criteria and were included in this review (Figure 1).

### 2.10. Risk of Bias

The risk of bias was evaluated by the reviewers (A.P., E.C.) using the Cochrane Collaboration’s two-part tool (Table 3) [25]. Ratings were assigned as “+” (low risk), “-” (high risk), or “?” (unclear risk) for each question in the checklist. If any disagreement due to risk of bias arose, the third reviewer (I.V.) was asked to collaborate.

### 2.11. Data Extraction and Collection

The authors (A.P., E.C.) individually extracted data and collected the following characteristics from each study:

- Study details: authors, year, and design.
- Patient characteristics: number, age range of patients, and type/number of treated teeth.
- Clinical outcomes: KTW, CAL, GR, CRC, and surgery time.
- Patient-centered outcomes (esthetics, pain, and discomfort) at baseline and at 12-months of follow-up.

Data were transformed as necessary to ensure consistency in the analysis.

The assessment of publications was based on their relevance and eligibility, following the PRISMA statement guidelines [19], as shown in Figure 1.

### 2.12. Data Items

(a) “Author”—name of the first author and year of publication.
(b) “Study design”—type of study conducted.
(c) “Baseline records”—baseline clinical parameters, surgery time and patient-centered outcomes.
(d) “Clinical parameters”—parameters used to evaluate tissues around the teeth with GR: KTW, CAL, GRD, and CRC.
(e) “Patient-centered outcomes”—outcomes related to esthetics, pain, and patient satisfaction.
(f) “Surgery time”—duration of gingival recession coverage.
(g) “Number of patients, Intervention”—number of patients treated, and the treatment protocols used in the test and control groups.
(h) “Follow-up”—duration of follow-up (in months) for both the test and control groups.
(i) “Treatment outcomes”—clinical parameters, surgery time, and patient-centered outcomes with values recorded at baseline and at the end of follow-up (i.e., KTW, CAL, GR, CRC, esthetics, pain, satisfaction, and duration of the surgical procedure of recession coverage).

The characteristics of the included studies are summarized in Table 4.

### 2.13. Study Design and Characteristics of Patients

All the four included studies [15,22,23,24] were split-mouth RCTs enrolling adults with bilateral gingival recession defects (Miller’s Class I, II, or RT1, 2). Two studies used the old classification of GR according to Miller [22,24]. Miller’s Class I is described as gingival recession up to the mucogingival junction (MGJ) without interdental bone loss, whereas Miller’s Class II includes gingival recession starting from MGJ and continuing further, also without interdental bone loss [26].

RT1 and RT2 recession types belong to the classification proposed by Cairo et al. [27]: RT1—gingival recession at the buccal tooth surface without interproximal attachment loss; RT2—interproximal attachment loss less than or equal to that on the buccal site. This classification was used by the other two studies included in this review [15,23].

### 2.14. Preoperative Procedures

All patients in the included studies [15,22,23,24] completed oral hygiene prophylaxis and received oral hygiene instructions prior to treatment. Specific preoperative instructions included:

Menezes et al. [23]: intake of 4 mg dexamethasone and 500 mg amoxicillin (1 h prior to surgery).

Harris et al. [24]: rinse with 0.12% chlorhexidine for 1 min.

### 2.15. Types of Interventions

In all included studies, the test group was treated with XCM in combination with CAF, whereas in the control group, CTG with CAF was used. The CTG was harvested from the palate. The authors used split-thickness [15,24] or split-full-split thickness [22,23] flap types. The flaps were extended by two vertical incisions to the mucogingival junction. Root planning with curettes and/or composite refining drills were reported by Menezes et al. [23] and McGuire et al. [15]. Additionally, 24% EDTA and sterile saline were applied on the exposed roots in the study by McGuire et al. [15]. The grafts were sutured at recession sites, followed by coronal flap repositioning and suturing. The donor sites in the CTG group were sutured as well. The types of sutures used were Silk 4-0 [23,24], Vicryl 5-0 [24], Plain gut 5-0, 6-0 [15], and Nylon 5-0 [23].

### 2.16. Postoperative Care

Sutures were removed 1–2 weeks postoperatively [15,22,23,24]. All participants were instructed to rinse their mouths with a 0.12% [19,20,22] or 0.2% [15,18] chlorhexidine solution for 2–3 weeks and to avoid brushing in the surgical area [15,22,23,24]. In Table 5, the types of prescribed anti-inflammatory drugs and antibiotics are reported.

### 2.17. Statistical Analysis

The meta-analysis was performed using SPSS statistical software (version 31.0, SPSS Inc., Chicago, IL, USA). The heterogeneity of the studies was tested using the Cochrane Q test, where I^2^ = 100% × (Q − df)/Q. Cohen’s d, a standardized measure of the effect size for continuous variables, was calculated. According to Cohen, (1988) the effect size 0.2 is considered small, 0.5 is considered medium, and 0.8 is considered large [28]. With only four studies, the power to detect publication bias was limited and was therefore not assessed. The statistical significance level for the observed differences between the groups was set at *p* ≤ 0.05.

## 3. Results

In a total of 98 patients, 248 teeth with bilateral recessions were treated (125 test/123 control). Patients’ ages ranged from 18 to 55 years. The main characteristics of the considered studies are described in Table 4, including author, year of publication, patients’ characteristics, and clinical measurements. Clinical parameters, such as KTW, CAL, and GR, were measured at baseline and at 12-months of follow-up and are reported in Table 6.

The summary of the changes between baseline and 12 months postoperatively is presented below (also see Table 6).

### 3.1. KTW

In all clinical trials, KTW was significantly augmented in the grafted teeth of both test and control groups [15,22,23,24]. However, Nahas et al. and Harris et al. [22,24] reported significant differences in tissue augmentation favoring the control group, whereas the other studies did not find significant differences between the two groups [15,23].

### 3.2. GR

A reduction in GR was shown in all clinical trials with significant differences between baseline parameters and at 12-months of follow-up [15,22,23,24]. Additionally, McGuire et al. [15] reported a significantly higher reduction of GR in the control group compared to the test group, whereas the remaining three studies did not report differences between the groups at 12-months of follow-up.

### 3.3. CAL

A significant gain in CAL was noted in all clinical trials in both groups. Regarding inter-group comparison, only McGuire et al. [15] reported a significant gain in CAL, again in favor of the control group.

### 3.4. CRC

McGuire et al. [15] reported significant differences in CRC rates between the test and control groups during follow-up, favoring the control group. Although the studies by Nahas et al. and Menezes et al. [22,23] did not find statistically significant inter-group differences, Harris et al. [24] did not calculate CRC rate in their study.

### 3.5. Surgery Time

All clinical trials reported a significant decrease in surgery time when XCM was used [15,22,23,24].

### 3.6. Patient-Centered Outcomes

The approach to patient-centered outcomes assessment for each study differed. A visual analog scale (VAS) (0–10, from the worst to the best score) was used in the studies conducted by Nahas et al. [22] and Harris et al. [24]. The Oral Health-Related Quality of Life (OHRQoL) (1–5, from very bad to very good) was applied in the study conducted by Menezes et al. [23]. McGuire et al. [15] analyzed the outcomes using ANCOVA, where the levels of satisfaction were categorized as “unsatisfied”, “satisfied”, and “very satisfied”.

Postoperative pain was assessed in three studies. In each of these studies, the test groups had significantly less postoperative pain than the control groups [15,22,24]. In the study conducted by Nahas et al. [22], esthetic outcomes were also assessed. The esthetic score showed no significant difference between the groups [22]. In the study conducted by Menezes et al. [23], the patient-centered outcomes were not analyzed by group, and the physical, psychological, and social domains significantly improved after each type of treatment. The study by Harris et al. [24] also measured dental hypersensitivity, with results favoring the test group.

**Figure 2:**
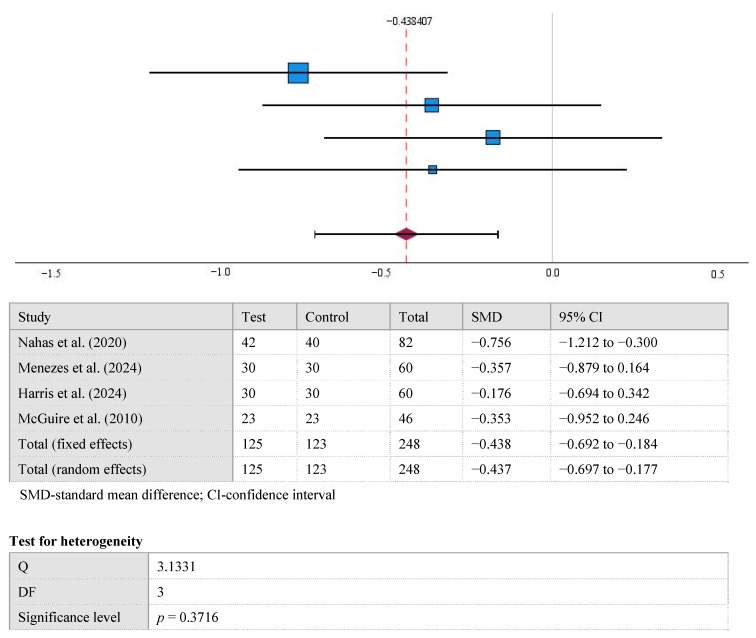
The difference in KTW values between the test and control groups at the 12-month follow-up [15,22,23,24].

The results displayed in the forest plot show a significant difference between the mean KTW values between the control and test groups at the 12-month follow-up (−0.438 [95% CI, −0.714 to −0.163], *p* < 0.002). Overall Cohen’s d of KTW after 12 months was 0.44 (medium). A high level of statistical homogeneity was observed in the meta-analysis of KTW (I^2^ = 4%, *p* > 0.05) (Figure 2).

**Figure 3:**
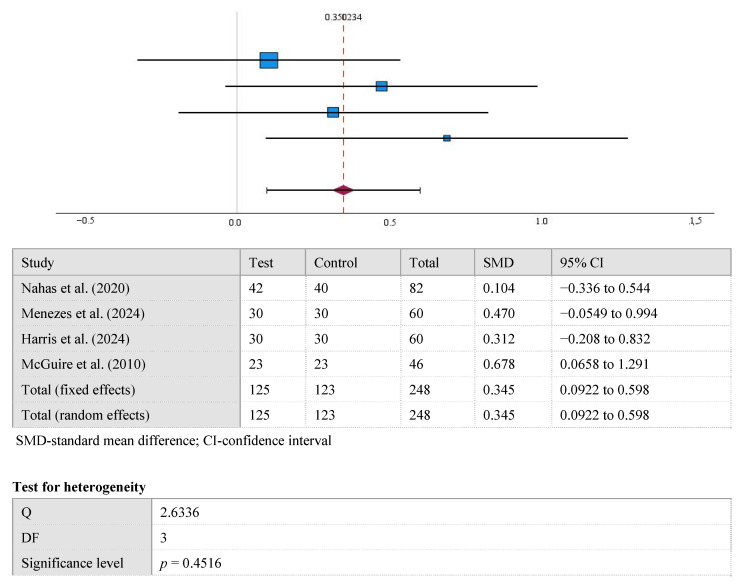
The difference in GR values between the test and control groups after the 12-month follow-up [15,22,23,24].

The results displayed in the forest plot show a significant difference between the mean GR values at the 12-month follow-up in the control and test groups (0.35 [95% CI, 0.098 to 0.602], *p* < 0.001). Overall, Cohen’s d for GR after 12 months was 0.35 (small). A high level of statistical homogeneity was observed in the meta-analysis of GR (I^2^ = 0%, *p* > 0.05) (Figure 3).

**Figure 4:**
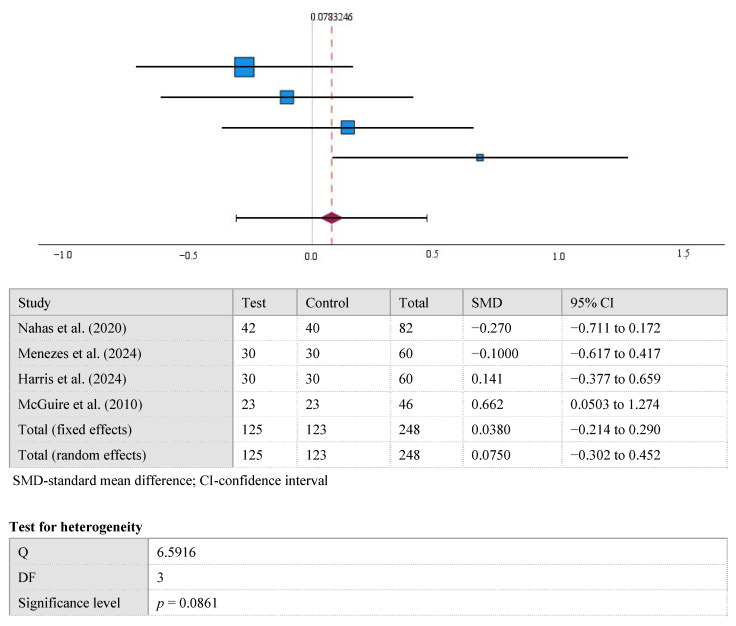
The difference in CAL values between the test and control groups after the 12-month [15,22,23,24].

The results displayed in the forest plot show no significant difference between the mean CAL values at the 12-month follow-up in the control and test groups (0.78 [95% CI, −0.305 to 0.461], *p* > 0.05). Overall, Cohen’s d for CAL was 0.08 (small). A high level of statistical heterogeneity was observed in the meta-analysis of CAL (I^2^ = 55%, *p* > 0.05) (Figure 4).

## 4. Discussion

In this systematic review and meta-analysis, XCM was investigated as a substitute for CTG harvested from the palate in the treatment of multiple gingival recessions. The comparison between the two approaches was based on the evaluation of clinical parameters (CRC, GRD, KTW, and CAL), as well as the patient-centered outcomes (pain, discomfort, and esthetics).

Both treatment options showed significant improvements across all parameters from baseline to follow-up, confirming their effectiveness in root coverage procedures. However, inter-group differences were noted at the 12-month follow-up, particularly in CRC, which is considered the primary clinical parameter for evaluating the success of gingival recession coverage [29]. In the analyzed studies that included CRC parameters, the rate of CRC was higher in the CTG group compared to the XCM group. Moreover, one of these studies reported statistically significant inter-group differences (99.3% in CTG vs. 88.5% in XCM [15]; *p* < 0.05).

McGuire et al. [15] highlighted anatomical differences in treatment outcomes between the maxilla and the mandible. Surgical procedures and subsequent healing processes are more complicated in the mandible due to muscle pull and decreased vascular supply. Therefore, they also provided CRC data for maxillary teeth only, where no significant difference between the groups was found (91.1% ± 19.6% CRC in the XCM group and 99.2% ± 3.6% in the CTG group). The comparable results were reported by Nahas et al. [22].

Similarly, Matoh et al. [30] reported findings consistent with our review: 100% of CRC in the CTG group versus 85% in the XCM group, supporting the non-inferiority of XCM in terms of root coverage.

The other clinical parameters, such as KTW, CAL, and GR, yielded mixed results. Among the reviewed studies, Nahas et al. [22] and Harris et al. [24] reported a statistically significant difference in KTW gain, favoring the CTG group. McGuire et al. [15] observed significant differences in GR reduction and CAL gain. However, no other studies reported statistically significant inter-group differences related to GR reduction, CAL gain, or KTW. These results are consistent with the study conducted by Cardaropoli et al. [31], who also reported no statistically significant inter-group differences in GR, CAL, or KTW at the 12-month follow-up. Small differences in CAL and KTW between the test and control groups may indicate that both CTG and XCM are linked with adequate stability of the gingival tissues, successful treatment outcomes, and good prognosis of recession coverage.

The superiority of CTG observed in some studies may be explained by its biological composition. According to Menezes et al. [23], CTG contains living cells, blood vessels, collagen, and other constituents, whereas XCM is composed solely of collagen. These compositional differences may influence the healing process and cell regeneration. The study conducted by Ashurko et al. [32] linked CTG with better soft tissue regeneration around implants compared to XCM.

To address limitations related to XCM composition, CAF is often preferred when using XCM. By placing the incision away from the recession area, CAF enhances vascularization and tissue nutrition, thereby accelerating the healing process [23]. However, alternative approaches such as TUN, which is less invasive, have also been evaluated. For example, in the study conducted by Tavelli et al. [33], TUN was compared with CAF, and no statistically significant differences were noted in KTW, CRC, mean root coverage, or root coverage esthetics. Cieślik-Wegemund et al. [34] compared XCM and CTG used with TUN and found similar KTW and CAL gains, although CRC and GR outcomes favored CTG. In contrast to our findings, postoperative pain was higher in the XCM group than in the CTG group.

Regarding the patient-centered outcomes, they consistently favored XCM. The majority of analyzed clinical trials reported reduced pain and discomfort, shorter recovery time, and lower analgesic intake in the XCM group. These findings can be attributed to the absence of a second surgery site required for CTG harvesting. McGuire et al. [15] clearly documented the additional pain associated with the donor site in CTG harvesting.

In terms of alternative biomaterials, XCM has shown superior outcomes. Jepsen et al. [35] compared the results of their clinical trial (XCM + CAF) with those of Harris et al. [36], in which acellular dermal matrix (ADM) was used in combination with CAF. The XCM + CAF group demonstrated greater long-term stability in root coverage outcomes, whereas only 32% of patients in the ADM group maintained stable outcomes.

A minimum 12-month follow-up period was set as an inclusion criterion in this review to ensure the clinical reliability and long-term relevance of results. However, a shorter 6-month follow-up may still be considered. The outcomes gathered in the 6-month follow-up reported by Tonetti et al. [37] were consistent with those in our review: CRC was higher in the CTG + CAF group compared to the XCM + CAF group, and both GR reduction and KTW gain were also greater in the CTG + CAF group.

In the study conducted by Jepsen et al. [18], results at 6-month follow-ups and at 3 years were compared, and a high correlation in CRC outcomes over time was observed. No significant differences were noted between the 6-month and 3-year follow-ups. Therefore, it was suggested that using the results acquired at the 6-month follow-ups, practitioners could predict long-term results. However, this assumption must be taken with caution, as only 40% of the original patients were examined at the 3-year follow-up in the above-mentioned study [18].

The present systematic review has several limitations that should be acknowledged.

First, a limited number of cases were available for this meta-analysis, as only four studies met the inclusion criteria. However, it is important to note that the selected clinical trials matched the strict requirements for search and data extraction. Most of them carried a low risk of bias. All studies included in this review had a split-mouth design for comparison of the treatment outcomes using two different materials. This approach reduces variability of the estimated treatment, ensuring that test and control sites operated under equal conditions and received the same postoperative care. The split-mouth design also shows that the tissues presented the same biological behavior in both treatment groups [23].

Second, the gingival phenotype was not evaluated prior to or after the surgeries. Different gingival phenotypes may respond variably to surgical procedures due to differences in blood flow rate and soft tissue composition [38]. Despite the possible differences in gingival phenotype, the clinical outcomes were better in the control groups of the selected studies.

The heterogeneity in measuring patient-centered outcomes could be considered another limitation, as there were variations in the assessment tools used (ANCOVA, OHRQoL) and in the follow-up time points. Nevertheless, the qualitative features of the measurements were similar and generally showed better tolerance of the XCM + CAF grafts. A high level of statistical heterogeneity among the studies observed in the meta-analysis of CAL and the relatively small number of treated sites could also interfere with the final outcomes. Moreover, publication bias (Egger’s test) could not be evaluated due to the small number of studies included in this review.

Although a follow-up period of 12 months is sufficient to observe tissue response to the interventions, further long-term observations would be valuable to confirm the stability of tissues around the teeth after the treatment of gingival recession.

The newer generations of XCM, such as cross-linked or hybrid types with a greater number of treated sites, should also be investigated in clinical trials, taking into consideration important factors such as material costs, strict temperature storage requirements, and shelf-life limitations.

## 5. Conclusions

Within the limitations of the current systematic review and meta-analysis, it can be concluded that CTG + CAF remains the gold standard for root coverage procedures. However, XCM could be recommended in several cases, such as for elderly patients, for those with specific medical conditions, for individuals lacking subepithelial connective tissue due anatomical factors, or when CTG is refused.

## Figures and Tables

**Table 1:** PICOT table.

Component	Description
Population (P)	Adult patients diagnosed with multiple gingival recessions undergoing root coverage treatments
Intervention (I)	Soft tissue augmentation using XCM
Comparison (C)	Soft tissue augmentation using CTG
Outcome (O)	Primary: KTW, GR, CAL, and CRC Secondary: patient complaints, discomfort, overall satisfaction, and surgery time
Time (T)	12 months after surgery

XCM—xenogeneic collagen matrix; CTG—connective tissue graft; KTW—keratenized tissue width; GR—gingival recession depth; CAL—clinical attachment level; CRC—complete root coverage.

**Table 2:** Publication search strategy.

Search #	Search Words
1	“Collagen matrix” [All Fields] OR “Xenogeneic collagen matrix” [All Fields] OR “CM” [All Fields] OR “XCM” [All Fields]
2	“Connective tissue graft” [All Fields] OR “CTG” [All Fields] OR “Subepithelial connective tissue graft” [All Fields] OR “SCTG” [All Fields] OR “Palatal connective tissue graft” [All Fields]
3	“Coronally advanced flap” [All Fields] OR “CAF” [All Fields]
4	“Multiple recessions” [All Fields] OR “Bilateral recessions” [All Fields] OR “Recessions” [All Fields] OR “RT1 recessions” [All Fields] OR “Miller’s Class 1” [All Fields] OR “RT2 recessions” [All Fields] OR “Miller’s Class 2” [All Fields]
5	#1 AND #2 AND #3 AND #4

CM—collagen matrix; SCTG—subepithelial connective tissue graft; CAF—coronally advanced flap.

**Table 3:** Risk of bias evaluation.

Study	Random Sequence Generation	Allocation Concealment	Blinding of Outcome Assessment	Incomplete Outcome Data	Selective Reporting	Other Sources of Bias
Nahas et al. (2020) [22]	+	+	+	+	+	+
Menezes et al. (2024) [23]	+	?	+	+	+	+
Harris et al. (2024) [24]	+	?	?	+	+	+
McGuire et al. (2010) [15]	+	+	+	+	+	+

**Table 4:** Characteristics of the included studies.

Study	N—Number of Patients, n—Total Number of Treated Teeth (Test/Control), Type of Teeth	Age of Patients Mean Age (SD)	Patient-Centered Outcomes	Clinical Parameters Investigated
Nahas et al. (2020) [22]	N = 15 n = 82 (42/40) Maxillary 100% (canines, first and second premolars)	32.7 (8.1)	Postoperative pain, DH (VAS)	GRD, KTW, CAL, CRC, PD, Surgery time
Menezes et al. (2024) [23]	N = 30 n = 60 (30/30) Maxillary 100% (canines, first and second premolars)	30.3 (6)	Quality of life (physical, social, and psychological domains) (OHRQoL)	GRD, PD, CAL, BOP, CRC, GT, KTW, Surgery time
Harris et al. (2024) [24]	N = 30 n = 60 (30/30) Not specified	34.8 (6.2)	Pain sensitivity (VAS)	GRD, PD, CAL, GRW, KTW, Surgery time
McGuire et al. (2010) [15]	N = 23 n = 46 (23/23) Not specified	43.7 (12)	Discomfort assessment (ANCOVA)	GRD, CAL, KTW, PD, GRW, BOP, CRC

GRD—gingival recession depth, GRW—recession width; KTW—keratinized tissue width; CAL—clinical attachment level; BOP—bleeding on probing; PD—probing depth; CRC—complete root coverage; GT—gingival thickness; GRW—gingival recession width; DH—dentin hypersensitivity, VAS—visual analog scale; OHRQoL—The Oral Health-Related Quality of Life; ANCOVA—analysis of covariance.

**Table 5:** Description of prescribed anti-inflammatory drugs and antibiotics.

Studies	Prescribed Anti-Inflammatory Drugs	Prescribed Antibiotics
Nahas et al. (2020) [22]	100 mg nimesulide (twice a day for 3 days)	-
Harris et al. (2024) [24]	Zerodol-SP (twice a day for 3 days)	500 mg amoxicillin twice daily for 3 days
Menezes et al. (2024) [23]	100 mg nimesulide (every 12 h for 3 days), 500 mg dipyrone (every 6 h for 3 days)	500 mg amoxicillin (every 8 h for 1 day)
McGuire et al. (2010) [15]	ibuprofen or hydrocodone	100 mg doxycycline daily (for 10 days)/amoxicillin (for 7 days)

**Table 6:** Clinical and patient-centered outcomes at baseline and at 12-months follow-up.

Study	Clinical Parameters at Baseline (Test vs. Control) Mean (SD)	Clinical Parameters After 12 Months (Test vs. Control) Mean (SD)	Operating Time (Test vs. Control) Mean (SD)	Patient-Centered Outcomes (Test vs. Control) Mean (SD)
Nahas et al. (2020) [22]	KTW: 2.2 (1.0) vs. 2.1 (1.0) GR: 2.7 (1.1) vs. 2.8 (1.1) CAL: 3.8 (1.1) vs. 4.0 (1.2)	KTW: 2.5 (0.7) vs. 3.2 (1.1) * GR: 0.6 (1.0) vs. 0.5 (0.9) CAL: 1.9 (1.0) vs. 2.2 (1.2) CRC: 60% vs. 68%	31.3 (4.3) vs. 47.7 (6.1) *	Postoperative pain: 1.34 (1.63) vs. 2.73 (2.39) * DH: 2.4 (3.6) vs. 2.1 (3.2)
Menezes et al. (2024) [23]	KTW: 3.3 (1.3) vs. 3.3 (1.42) GR: 2.4 (0.98) vs. 2.7 (1.24) CAL: 3.9 (1.29) vs. 4.2 (1.45)	KTW: 4 (1.34) vs. 4.5 (1.42) GR: 0.62 (0.79) vs. 0.3 (0.53) CAL: 1.9 (0.86) vs. 2 (1.10) CRC: 66.7% vs. 70%	45.6 (6.9) vs. 75.5 (11.4) *	Not specified by control and test group Physical domains (t0–t12): 21.3 (0.88)–27.6 (0.51) * Social domains (t0–t12): 18.5 (0.82)–22.4 (0.37) * Psychological domains (t0–t12): 18.1 (0.84)–22.2 (0.42) *
Harris et al. (2024) [24]	KTW: 2.2 (0.2) vs. 2.1 (0.2) GR: 3.4 (0.8) vs. 3.5 (0.8) CAL: 5.4 (1.03) vs. 5.4 (1.02)	KTW: 3.2 (0.8) vs. 3.9 (5.5) * GR: 0.3 (0.4) vs. 0.2 (0.2) CAL: 2.4 (0.7) vs. 2.3 (0.7)	Not specified but mentioned that in the test group, the duration of surgery was reduced.	Fewer pain reports in the test group. Reported decrease in dental hypersensitivity after intervention in both groups.
McGuire et al. 2010) [15]	KTW: 2.44 (1.02) vs. 2.78 (1.35) GR: 3.14 (0.23) vs. 3.20 (0.35) CAL: 4.40 (0.61) vs. 4.50 (0.61)	KTW: 3.59 (1.04) vs. 3.98 (1.13) GR: 0.37 (0.71) vs. 0.02 (0.1) * CAL: 2.13 (0.90) vs. 1.63 (0.54) * CRC: 88.5% vs. 99.3% *	Not mentioned	Postoperative pain: After 1 week 8 vs. 9 + 3 (donor site); equivalent pain—6 * After 4 weeks 3 vs. 7 + 5 (donor site); equivalent pain—10 *

KTW—keratinized tissue width; GR—gingival recession depth; CAL—clinical attachment level, CRC—complete root coverage, DH—dentin hypersensitivity. *—Significant *p* value (*p* < 0.05).
